# Reducing motion artifacts in the aorta: super-resolution deep learning reconstruction with motion reduction algorithm

**DOI:** 10.1007/s11604-025-01849-8

**Published:** 2025-08-09

**Authors:** Koichiro Yasaka, Rin Tsujimoto, Rintaro Miyo, Osamu Abe

**Affiliations:** Department of Radiology, The University of Tokyo Hospital, 7-3-1 Hongo, Bunkyo-Ku, Tokyo 113-8655 Japan

**Keywords:** Aorta, Motion artifact, Computed tomography, Deep learning reconstruction, Super-resolution

## Abstract

**Purpose:**

To assess the efficacy of super-resolution deep learning reconstruction (SR-DLR) with motion reduction algorithm (SR-DLR-M) in mitigating aorta motion artifacts compared to SR-DLR and deep learning reconstruction with motion reduction algorithm (DLR-M).

**Materials and methods:**

This retrospective study included 86 patients (mean age, 65.0 ± 14.1 years; 53 males) who underwent contrast-enhanced CT including the chest region. CT images were reconstructed with SR-DLR-M, SR-DLR, and DLR-M. Circular or ovoid regions of interest were placed on the aorta, and the standard deviation of the CT attenuation was recorded as quantitative noise. From the CT attenuation profile along a line region of interest that intersected the left common carotid artery wall, edge rise slope and edge rise distance were calculated. Two readers assessed the images based on artifact, sharpness, noise, structure depiction, and diagnostic acceptability (for aortic dissection).

**Results:**

Quantitative noise was 7.4/5.4/8.3 Hounsfield unit (HU) in SR-DLR-M/SR-DLR/DLR-M. Significant differences were observed between SR-DLR-M *vs.* SR-DLR and DLR-M (*p* < 0.001). Edge rise slope and edge rise distance were 107.1/108.8/85.8 HU/mm and 1.6/1.5/2.0 mm, respectively, in SR-DLR-M/SR-DLR/DLR-M. Statistically significant differences were detected between SR-DLR-M *vs.* DLR-M (*p* ≤ 0.001 for both). Two readers scored artifacts in SR-DLR-M as significantly better than those in SR-DLR (*p* < 0.001). Scores for sharpness, noise, and structure depiction in SR-DLR-M were significantly better than those in DLR-M (*p* ≤ 0.005). Diagnostic acceptability in SR-DLR-M was significantly better than that in SR-DLR and DLR-M (*p* < 0.001).

**Conclusions:**

SR-DLR-M provided significantly better CT images in diagnosing aortic dissection compared to SR-DLR and DLR-M.

## Introduction

Acute aortic dissection is a life-threatening condition. Studies have shown that mortality rates following out-of-hospital cardiac arrest due to acute aortic dissection were 100% [1–3]. Even with open surgical treatment, the 30-day mortality and in-hospital mortality for patients with acute Stanford type A aortic dissection are 9.2% and 11%, respectively [4]. Early diagnosis and treatment are crucial for patient outcomes, and CT plays a vital role in diagnosing and classifying acute aortic dissection [3, 5]. However, motion artifacts are commonly observed in the aorta, mimicking the intimal flap of the aortic dissection [6]. Due to differences in experience, this can result in the misdiagnosis of aortic dissection [5, 7]. Researchers have attempted to mitigate these artifacts using electrocardiography-assisted CT [8] or scanning techniques with faster gantry rotations and higher helical pitches [9]. Nevertheless, electrocardiography-assisted CT scans are time-consuming, and scans with faster gantry rotations and higher helical pitches are associated with image quality degradation [10–12].

Since mid-2010s, deep learning has garnered significant attention in the field of radiology [13, 14]. Deep learning has proven beneficial for radiologists in various aspects, including lesion detection [15, 16], differential diagnosis [17], and image quality enhancement [18]. Deep learning reconstruction (DLR) algorithm is particularly noteworthy in reducing image noise [19], contributing to improved lesion depiction and detection performance without extending reconstruction time [20–22], unlike iterative reconstruction [23]. Consequently, DLR has gained widespread acceptance in clinical practice. Recently, super-resolution DLR (SR-DLR) has emerged as a new algorithm. SR-DLR utilizes high spatial resolution CT images [24] as reference data during training, enabling the achievement of both reduced image noise and enhanced spatial resolution [25–27]. In addition to SR-DLR and DLR, a novel deep learning-based reconstruction technology has been introduced that mitigates motion artifacts. Based on these advancements, we hypothesized that the combination of these technologies would empower less-experienced readers to evaluate the aorta in chest CT with greater confidence.

The aim of this study was to evaluate the efficacy of combining the SR-DLR and motion reduction algorithm in enhancing image quality compared to employing SR-DLR alone or DLR with motion reduction algorithm.

## Materials and methods

This retrospective study was approved by our Institutional Review Board, which waived the requirement for obtaining written informed consent from patients.

### Patients

Patients who underwent contrast-enhanced CT involving the chest region in March 2025 were consecutively included in this study (*n* = 93). One patient whose CT images exhibited severe metal artifacts was excluded. Additionally, six patients were randomly selected and excluded from the main analyses to utilize their CT images for training purpose prior to a subjective image evaluation (as described subsequently).

### CT imaging and reconstruction

All patients underwent CT examination including the chest region with a single scanner (Aquilion ONE INSIGHT Edition [Canon Medical Systems; Tokyo, Japan]). The CT scan parameters were as follows: tube voltage, 120 kVp; tube current, automatic tube current modulation with a standard deviation of 13.0 for a 5 mm slice thickness; scan mode, helical; helical pitch, 0.813; gantry rotation time, 0.5 s; beam collimation, 40 mm; and detector collimation, 0.5 mm. Contrast material with iodine dose of 600 mgI/kg was administered intravenously via the antecubital vein using a power injector. Chest CT images were reconstructed with the following three algorithms developed by Canon Medical Systems:

- Precise IQ Engine with CLEAR Motion (SR-DLR-M),
- Precise IQ Engine without CLEAR Motion (SR-DLR), and.
- Advanced intelligent Clear-IQ Engine with CLEAR Motion (DLR-M).

Precise IQ Engine (Canon Medical Systems) and Advanced intelligent Clear-IQ Engine (Canon Medical Systems) are super-resolution deep learning reconstruction and deep learning reconstruction algorithms, respectively. CLEAR Motion (Canon Medical Systems) is a deep learning-based motion reduction algorithm employed during the image reconstruction process. The pixel resolution for rows/columns was 1024/1024, 1024/1024, and 512/512 for SR-DLR-M, SR-DLR, and DLR-M, respectively. The field of view (350 mm, adjusted to the patient’s body size) and slice thickness/interval (3 mm/3 mm) remained consistent across the three reconstruction algorithms. Based on the vendor’s recommendation, reconstruction strength level of 2, which corresponds to “Standard” setting in previous Canon CT systems, was used for SR-DLR-M, SR-DLR, and DLR-M.

### Concepts of SR-DLR, DLR, and motion reduction algorithms

In the development of SR-DLR [28] and DLR [29], scan data were acquired with high spatial resolution CT equipped with 0.25-mm collimation detector [24] and conventional CT equipped with 0.5 mm collimation detector, respectively. From these scan data, images were reconstructed with model-based iterative reconstruction [23] and were served as reference data for supervised training of deep learning models. This approach enables the achievement of noise reduction for both algorithms and the enhancement of spatial resolution for the SR-DLR algorithm.

Motion reduction algorithm (CLEAR motion) (https://www.google.com/url?sa=t&rct=j&q=&esrc=s&source=web&cd=&ved=2ahUKEwjc97qpvJGOAxXXqFYBHQfdMqkQFnoECA8QAQ&url=https%3A%2F%2Fch-de.medical.canon%2Fwp-content%2Fuploads%2Fsites%2F18%2F2025%2F03%2Fem_visionsblog_20250325_01_03_CLEAR-Motion-White-Paper-Deep-Learning-Raw-Data-based-Motion-Correction.pdf&usg=AOvVaw3nPTVRIxyCh_pecrDpzb6l&opi=89978449) employs projection data from multiple angles to correct for the motion of the scanned object. While projection data from the opposite side are theoretically identical, there can be discrepancies due to motion within the scanned object. The motion reduction algorithm employs a deep learning to estimate patterns of motion, which is trained on a substantial dataset containing such differences.

### Objective image evaluation

**Fig. 1:**
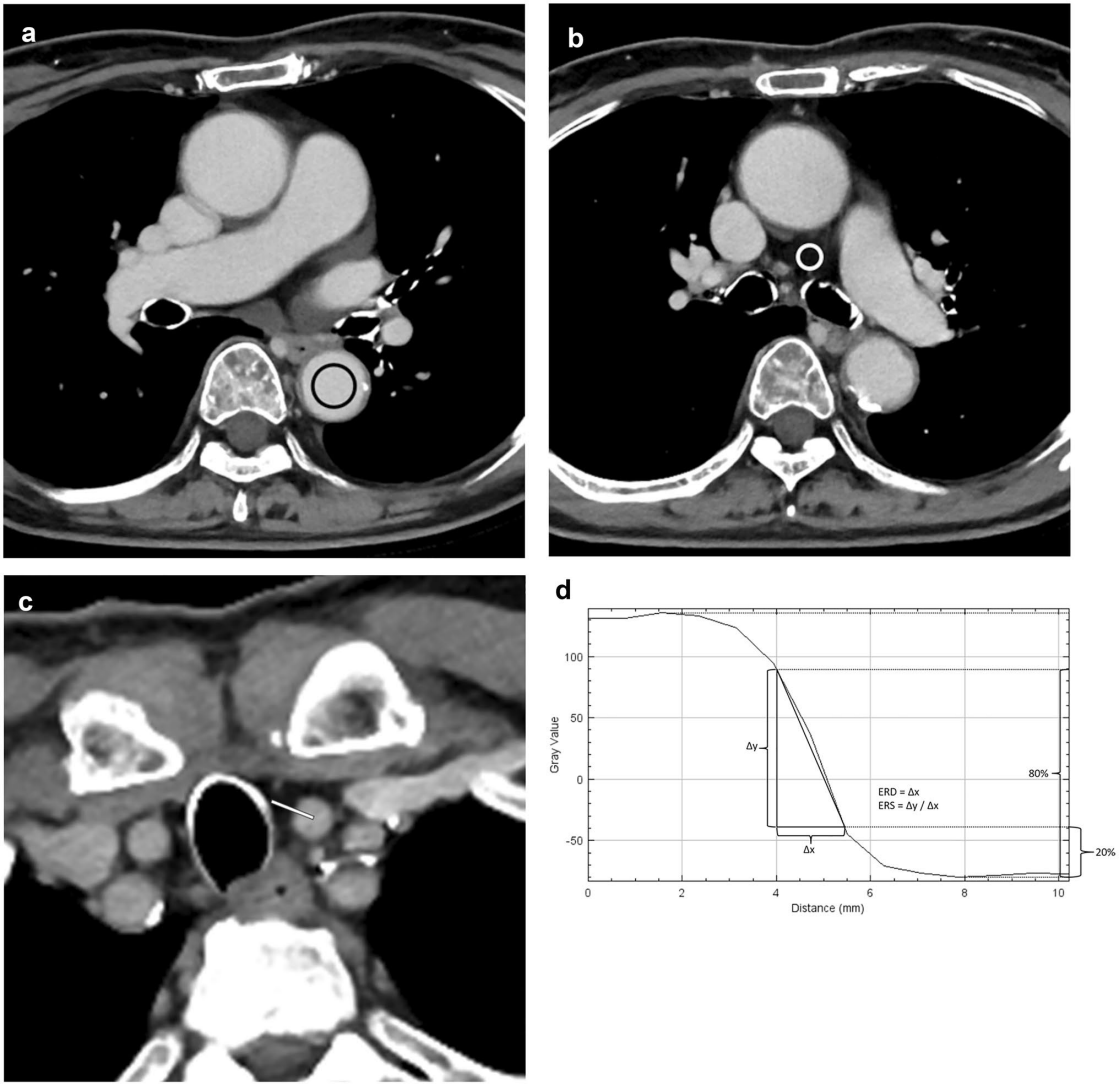
Circular or ovoid regions of interest were placed on (**a**) the aorta (black circle) and (**b**) mediastinal fat (white circle). A linear region of interest (white line) was placed transversely across the wall of the left common carotid artery in a rectangular manner (**c**). From the CT attenuation profile along the linear region of interest, edge rise distance (ERD) and edge rise slope (ERS) were calculated with setting the reference points on the curve with 20%–80% values (**d**)

A radiologist (Radiologist A, with imaging experience of 15 years) conducted objective image evaluations on Image J (https://imagej.net/ij/). Circular or ovoid regions of interest (ROIs) were placed on the descending aorta at the slice level of the main pulmonary trunk (Fig. 1a) and on the mediastinal fat (Fig. 1b) in SR-DLR-M, and they were copied to SR-DLR and DLR-M. The diameter of the ROI on the aorta was set at about 15 mm. The mean and standard deviation of CT attenuation within the ROI were recorded. The contrast-to-noise ratio (CNR) was subsequently calculated using the following formula:

$${\text{CNR}}\, = \,\left( {{\text{CT}}_{{{\text{AORTA}}}} {-}{\text{CT}}_{{{\text{FAT}}}} } \right) \, / \, \left( {{\text{SD}}_{{{\text{AORTA}}}}^{{2}} \, + \,{\text{SD}}_{{{\text{FAT}}}}^{{2}} } \right)^{{{1}/{2}}}$$

where CT_AORTA_, CT_FAT_, SD_AORTA_, and SD_FAT_ represent the mean CT attenuation of the aorta, the mean CT attenuation of the mediastinal fat, the standard deviation for CT attenuation of the aorta, and standard deviation for CT attenuation of the mediastinal fat, respectively. SD_AORTA_ was utilized as an indicator of quantitative image noise.

A linear ROI was placed across the left common carotid artery at a right angle (Fig. 1c) in SR-DLR-M and was copied to SR-DLR and DLR-M. This linear ROI was positioned to avoid arterial wall calcification. We selected the left common carotid artery instead of the aorta due to the following reasons; motion artifacts can affect the measurements in the ascending aorta, the aortic arch does not run perpendicular to the slice plane, and most parts of the descending aorta are surrounded by the lungs for which CT attenuation is far below the lower limit of the mediastinal window setting. From the CT attenuation profile along the linear ROI, edge rise distance (ERD) and edge rise slope (ERS) were calculated (Fig. 1d).

All quantitative measurements were performed twice, and the averaged values were used for the analyses.

### Subjective image evaluation

Two radiology residents (reader 1 and 2, with imaging experience of 5 and 2 years, respectively) were involved in the subjective image evaluation. Radiologist A randomized all the image datasets before the evaluations by the readers. The images were configured to appear in the same size on the monitor. Both readers were blinded to the background information. Using Image J, they independently scored the image datasets for the following evaluation items:

* Degree of artifacts in the ascending aorta (4 = no or minimal artifacts, 3 = mild artifacts, 2 = moderate artifacts, and 1 = severe artifacts interfering with diagnosis).
* Image noise (4 = no or minimal noise, 3 = mild noise, 2 = moderate noise, and 1 = severe noise interfering with diagnosis).
* Sharpness (4 = sharp, 3 = slightly blurred for some regions, 2 = moderately blurred, and 1 = blurred for most regions).
* Depiction of structures (aorta and mediastinal lymph nodes) (4 = clear, 3 = unclear for some regions, 2 = moderately unclear, and 1 = unclear for most regions).
* Diagnostic acceptability in the evaluations of aortic dissection (4 = Optimal, 3 = slight image degradation is seen but acceptable, 2 = somewhat unsuitable for diagnosis, and 1 = unacceptable) (this evaluation item can be affected by several factors, such as image noise, sharpness, and the degree of artifacts in the ascending aorta).
* The presence of the aortic dissection (2 = present, 1 = probably present, 0 = unsure, −1 = probably absent, −2 = absent).

The reference standard for the presence of the aortic dissection was established by Radiologist A based not only on image findings but also on the patient’s background information.

### Statistics

Statistical analyses were performed with R 4.1.2. Scores in the objective image evaluation and the subjective image evaluation were compared between SR-DLR-M *vs.* SR-DLR or DLR-M using the paired *t* test and Wilcoxon signed-rank test, respectively. Due to the multiple comparisons (SR-DLR-M *vs.* SR-DLR and SR-DLR-M *vs.* DLR-M), the Bonferroni correction was applied, and *p* < 0.025 (= 0.050/2) was considered to indicate a statistically significant difference.

## Results

### Patients

In this study, 86 patients (mean age, 65.0 ± 14.1 years; 53 males and 33 females; mean body mass index, 21.6 ± 3.9 kg/m^2^) were included in the final analyses. CT dose index volume (mGy)/dose-length product (mGy·cm)/effective dose (mSv) were the following; 5.9/350.7/4.9 for neck and chest (*n* = 1), 7.2 ± 0.2/515.7 ± 29.4/7.7 ± 0.4 for neck to abdomen (*n* = 2), 8.0 ± 2.6/739.0 ± 278.9/11.1 ± 4.2 for neck to pelvis (*n* = 21), 11.9 ± 1.9/581.8 ± 92.3/8.7 ± 1.4 for chest to abdomen (*n* = 2), and 9.8 ± 2.8/711.7 ± 238.2/10.7 ± 3.6 for chest to pelvis (*n* = 60).

### Objective image evaluation

Results for the objective image evaluation are presented in Table 1. CT attenuation of the aorta in SR-DLR-M (157.2 Hounsfield unit [HU]) was significantly higher than that in DLR-M (152.5 HU) (*p* < 0.001). CT attenuation of the fat in SR-DLR-M (− 74.8 HU) was significantly higher than that in DLR-M (− 77.1 HU) (*p* < 0.001). Quantitative image noise in SR-DLR-M was 7.4 HU, which was significantly lower than that in DLR-M (8.3 HU) (*p* < 0.001) but significantly higher than that in SR-DLR (5.4 HU) (*p* < 0.001). CNR for the aorta in SR-DLR-M (23.9) was lower than that in SR-DLR (29.6) (*p* < 0.001) but was significantly improved compared with DLR-M (22.5) (*p* < 0.001).

**Table 1:** Results for the objective image evaluation

	SR-DLR-M	SR-DLR	DLR-M	SR-DLR-M *vs.* SR-DLR	SR-DLR-M *vs.* DLR-M
CT_Aorta_ (HU)	157.2 ± 20.5	157.4 ± 20.0	152.5 ± 20.2	0.643	< 0.001*
CT_Fat_ (HU)	− 74.8 ± 21.4	− 74.5 ± 21.2	− 77.1 ± 20.9	0.768	< 0.001*
Noise (HU)	7.4 ± 0.9	5.4 ± 0.4	8.3 ± 1.0	< 0.001*	< 0.001*
CNR	23.9 ± 5.9	29.6 ± 7.6	22.5 ± 5.1	< 0.001*	< 0.001*
ERS (HU/mm)	107.1 ± 29.7	108.8 ± 28.4	85.8 ± 22.8	0.376	< 0.001*
ERD (mm)	1.6 ± 0.5	1.5 ± 0.6	2.0 ± 0.7	0.193	< 0.001*

Comparisons were conducted using paired *t* tests

*SR-DLR-M* super-resolution deep learning reconstruction with motion reduction algorithm, *SR-DLR* super-resolution deep learning reconstruction, *DLR-M* deep learning reconstruction with motion reduction algorithm, *CT*_*Aorta*_ CT attenuation of the aorta, *HU* Hounsfield unit, *CT*_*Fat*_ CT attenuation of the fat, *CNR* contrast-to-noise ratio, *ERS* edge rise slope, *ERD* edge rise distance

^*^Statistically significant difference (p < 0.025 [= 0.050/2])

ERS of the artery in SR-DLR-M (107.1 HU/mm) was significantly higher than that in DLR-M (85.8 HU/mm) (*p* < 0.001). ERD of the artery in SR-DLR-M (1.6 mm) was significantly smaller than that in DLR-M (2.0 mm) (*p* < 0.001).

### Subjective image evaluation

**Fig. 2:**
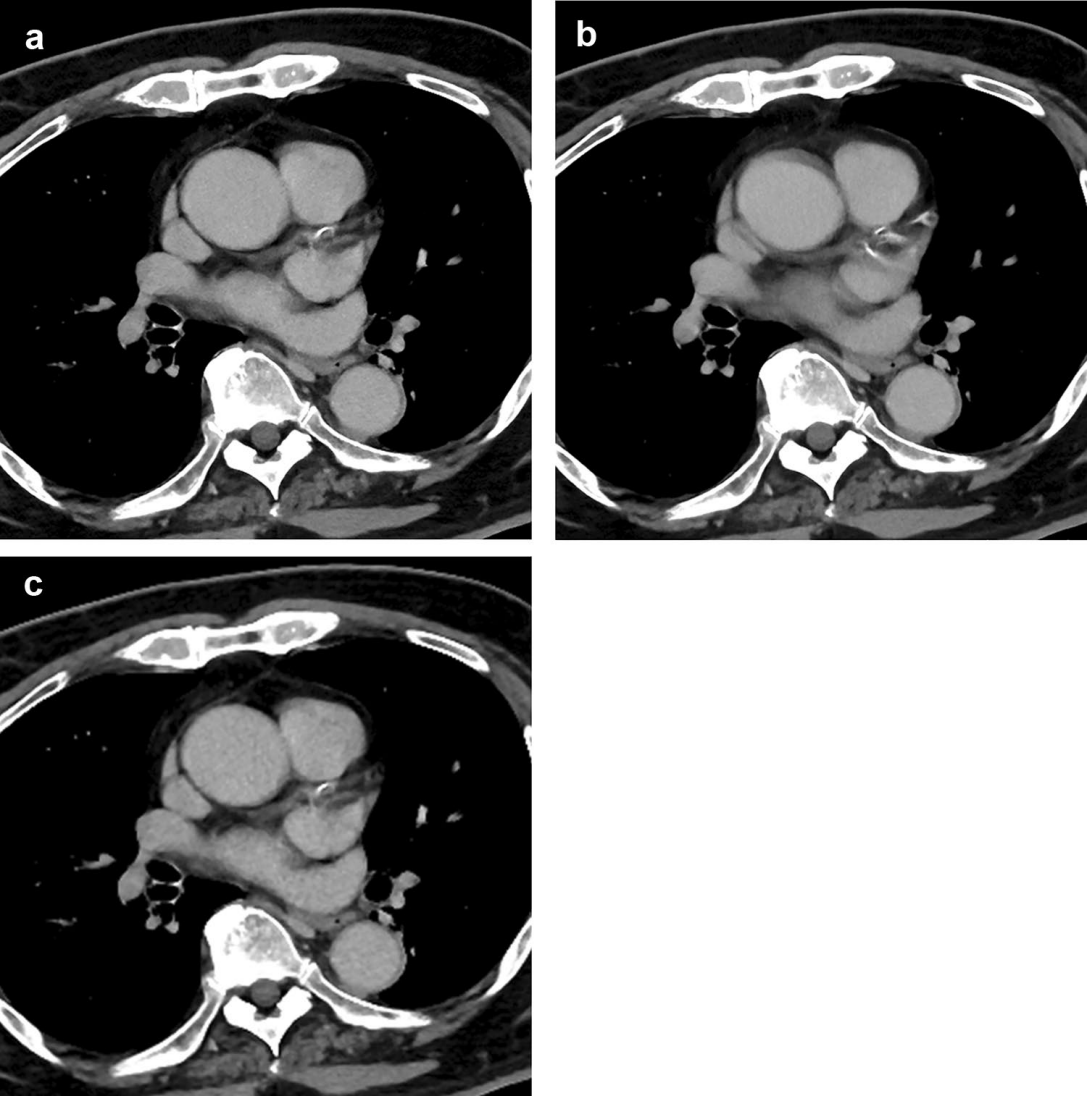
CT images of a 73-year-old male patient reconstructed using super-resolution deep learning reconstruction with motion reduction algorithm (SR-DLR-M) (**a**), super-resolution deep learning reconstruction (SR-DLR) (**b**), and deep learning reconstruction with motion reduction algorithm (DLR-M) (**c**). The artifact scores (SR-DLR-M/SR-DLR-DLR-M) were rated as 4 (no or minimal)/2 (moderate)/3 (mild) by both readers 1 and 2. The noise scores (SR-DLR-M/SR-DLR-DLR-M) were rated as 4 (no or minimal)/4/3 (mild) by reader 1 and 3/4/2 (moderate) by reader 2. Contrast-to-noise ratios (SR-DLR-M/SR-DLR-DLR-M) were 17.6/24.1/17.3

**Fig. 3:**
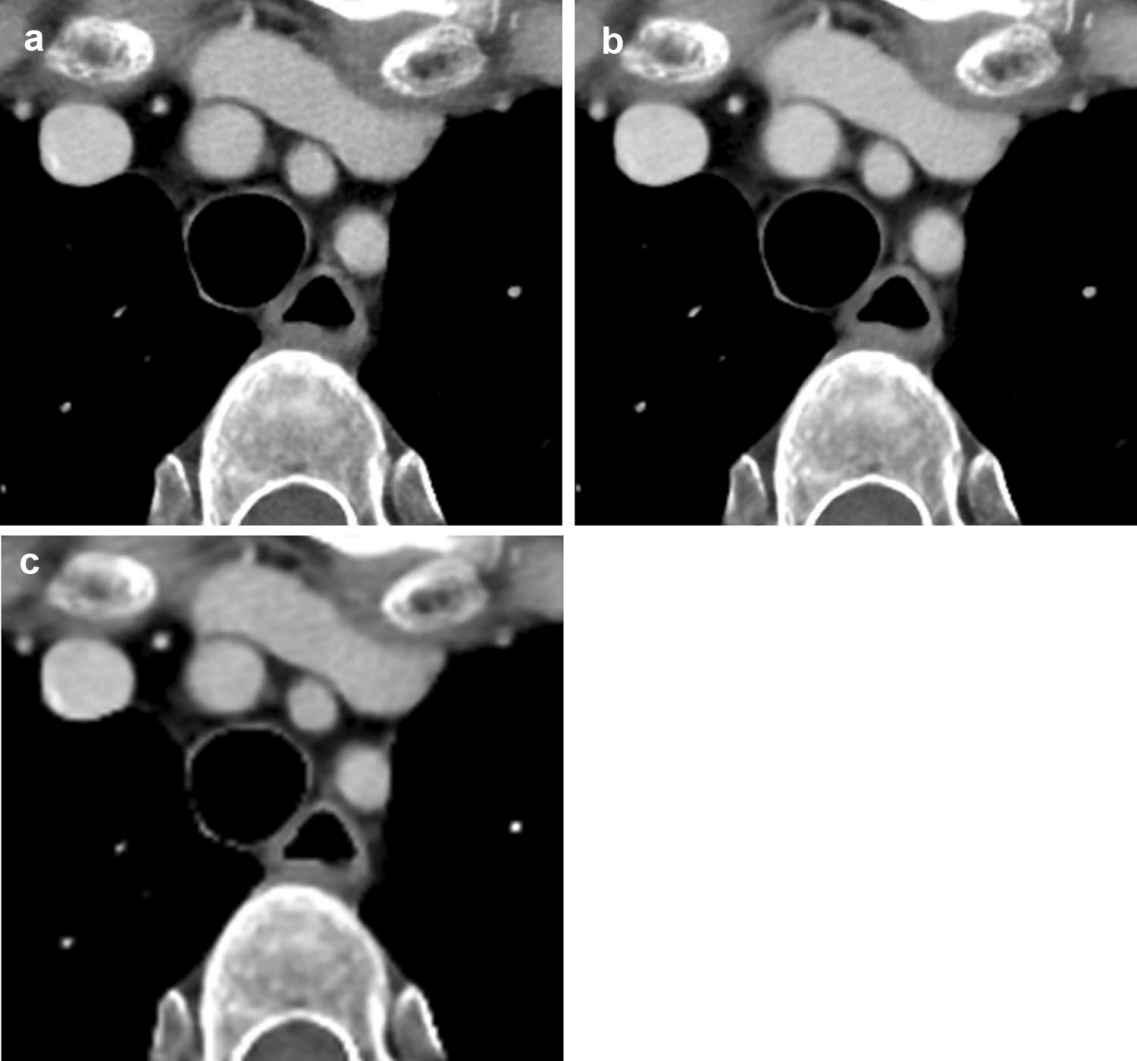
CT images of a 54-year-old female patient reconstructed using super-resolution deep learning reconstruction with motion reduction algorithm (SR-DLR-M) (**a**), super-resolution deep learning reconstruction (SR-DLR) (**b**), and deep learning reconstruction with motion reduction algorithm (DLR-M) (**c**). The sharpness scores (SR-DLR-M/SR-DLR-DLR-M) were 4 (sharp)/4/3 (slightly blurred for some regions) by both readers 1 and 2. Edge rise slope (Hounsfield unit/mm) and edge rise distance (mm) in SR-DLR-M/SR-DLR/DLR-M were 69.5/89.0/63.9 and 2.1/1.7/2.4, respectively

**Fig. 4:**
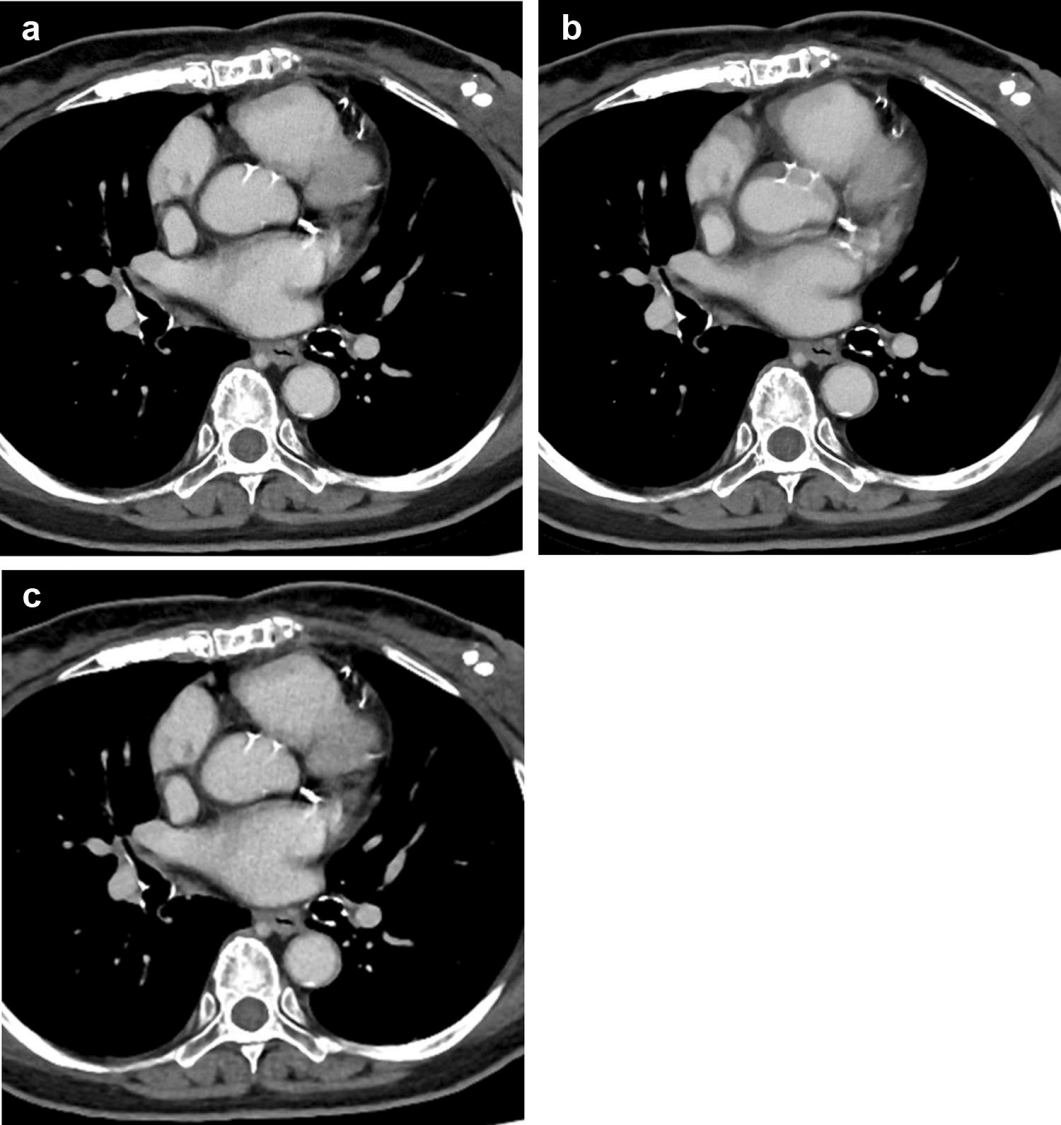
CT images of an 81-year-old female patient reconstructed using super-resolution deep learning reconstruction with motion reduction algorithm (SR-DLR-M) (**a**), super-resolution deep learning reconstruction (SR-DLR) (**b**), and deep learning reconstruction with motion reduction algorithm (DLR-M) (**c**). The artifact scores (SR-DLR-M/SR-DLR/DLR-M) were rated as 4 (no or minimal)/2 (moderate)/4 by reader 1 and 4/3 (mild)/4 by reader 2. The image noise scores (SR-DLR-M/SR-DLR/DLR-M) were rated as 4 (no or minimal)/4/2 (moderate) by both readers 1 and 2. The sharpness scores (SR-DLR-M/SR-DLR/DLR-M) were rated as 4 (sharp)/4/3 (slightly blurred for some regions) by reader 1 and 4/4/2 (moderately blurred) by reader 2. The diagnostic acceptability in the evaluations of aortic dissection scores (SR-DLR-M/SR-DLR/DLR-M) were 4 (optimal)/3 (slight image degradation is seen but acceptable)/2 (somewhat unsuitable for diagnosis) by reader 1 and 4/3/3 by reader 2

**Fig. 5:**
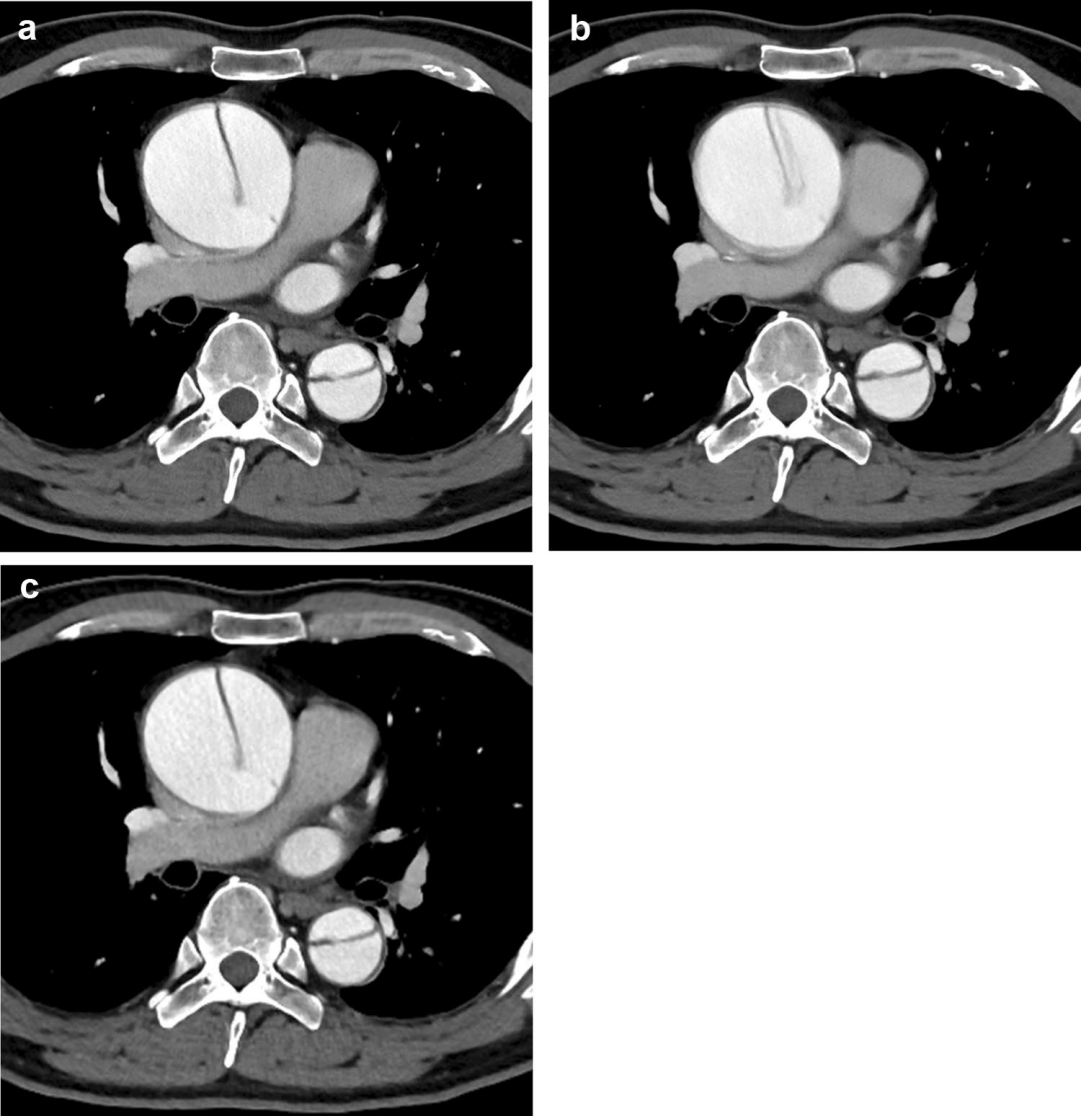
CT images of a 55-year-old male patient reconstructed with super-resolution deep learning reconstruction with motion reduction algorithm (SR-DLR-M) (**a**), super-resolution deep learning reconstruction (SR-DLR) (**b**), and deep learning reconstruction with motion reduction algorithm (DLR-M) (**c**) with Stanford type A aortic dissection. Motion artifacts of the flap in the ascending aorta, which are evident in SR-DLR (**b**), are diminished in both SR-DLR-M (**a**) and DLR-M (**c**). The image noise scores (SR-DLR-M/SR-DLR/DLR-M) were rated as 4 (no or minimal)/4/2 (moderate) by reader 1 and 4/4/3 (mild) by reader 2. The sharpness scores (SR-DLR-M/SR-DLR/DLR-M) were rated as 4 (sharp)/4/4 by reader 1 and 4/4/3 (slightly blurred for some regions) by reader 2. The diagnostic acceptability scores (SR-DLR-M/SR-DLR/DLR-M) were rated as 4 (optimal)/4/3 (slight image degradation is seen but acceptable) by reader 1 and 4/4/4 by reader 2

The results for the subjective image evaluation are summarized in Table 2. In SR-DLR, 69 (80.2%) and 62 (72.1%) of 86 patients were evaluated as having moderate or severe artifacts in the ascending aorta by readers 1 and 2, respectively. Scores for the artifacts of the ascending aorta in SR-DLR-M were significantly higher than those in SR-DLR (*p* < 0.001) (Fig. 2). Although a statistically significant difference was not observed, the scores for the artifacts tended to be higher in SR-DLR-M compared to DLR-M. Sharpness (Fig. 3), noise, depiction of cervical arteries, and depiction of lymph node in SR-DLR-M were rated as significantly improved compared to DLR-M by both readers (*p* ≤ 0.005). There were apparent differences in the score distribution of the sharpness and noise; readers rated scores of 4 for most patients in SR-DLR-M and SR-DLR but scores of 3 for most patients in DLR-M. Consequently, scores for acceptability for diagnosing aortic dissection in SR-DLR-M were significantly higher than those in SR-DLR and DLR-M by both readers (*p* < 0.001) (Fig. 4). CT images of a patient with aortic dissection are shown in Fig. 5. In patients without aortic dissection (*n* = 83), the number of patients assigned scores of 2/1/0/–1/–2 for the presence of aortic dissection by readers 1 and 2, respectively, were as follows: in SR-DLR-M, 0/0/0/7/76 and 0/0/0/1/82; in SR-DLR, 0/0/0/74/9 and 0/0/2/55/26; and in DLR-M, 0/0/0/5/78 and 0/0/0/0/83. There were statistically significant differences in those scores between SR-DLR-M *vs.* SR-DLR in both readers 1 and 2 (*p* < 0.001). On the contrary, there was no statistically significant differences in scores between SR-DLR-M *vs.* DLR-M for reader 1 (*p* = 0.530) and reader 2 (*p* = 1.000).

**Table 2:** Results of the subjective image evaluation

		SR-DLR-M	SR-DLR	DLR-M	SR-DLR-M *vs.* SR-DLR	SR-DLR-M *vs.* DLR-M
Artifact	Reader 1	57/19/10/0 (3.55)	8/9/48/21 (2.05)	51/27/8/0 (3.50)	< 0.001*	0.545
	Reader 2	67/14/5/0 (3.72)	11/13/60/2 (2.38)	58/28/0/0 (3.67)	< 0.001*	0.487
Sharpness	Reader 1	82/4/0/0 (3.95)	85/1/0/0 (3.99)	39/46/1/0 (3.44)	0.233	< 0.001*
	Reader 2	82/3/1/0 (3.94)	83/3/0/0 (3.97)	13/37/36/0 (2.73)	0.572	< 0.001*
Noise	Reader 1	76/10/0/0 (3.88)	82/4/0/0 (3.95)	1/43/42/0 (2.52)	0.092	< 0.001*
	Reader 2	73/12/1/0 (3.84)	79/7/0/0 (3.92)	1/17/68/0 (2.22)	0.133	< 0.001*
Depiction of cervical arteries	Reader 1	83/3/0/0 (3.97)	84/2/0/0 (3.98)	71/15/0/0 (3.83)	0.773	0.005*
	Reader 2	85/1/0/0 (3.99)	82/4/0/0 (3.95)	32/54/0/0 (3.37)	0.233	< 0.001*
Depiction of lymph node	Reader 1	79/6/1/0 (3.91)	80/6/0/0 (3.93)	44/40/2/0 (3.49)	0.565	< 0.001*
	Reader 2	82/4/0/0 (3.95)	78/8/0/0 (3.91)	15/57/14/0 (3.01)	0.182	< 0.001*
Diagnostic acceptability	Reader 1	55/21/10/0 (3.52)	8/10/51/17 (2.10)	18/60/8/0 (3.12)	< 0.001*	< 0.001*
	Reader 2	73/13/0/0 (3.85)	13/54/18/1 (2.92)	21/65/0/0 (3.24)	< 0.001*	< 0.001*

The patient counts for each score (4/3/2/1) are presented. In parentheses, mean scores are shown. Comparisons were conducted with the Wilcoxon signed-rank test

*SR-DLR-M* super-resolution deep learning reconstruction with motion reduction algorithm, *SR-DLR* super-resolution deep learning reconstruction, *DLR-M* deep learning reconstruction with motion reduction algorithm

^*^Statistically significant difference (p < 0.025 [= 0.050/2])

## Discussion

CT images of the aorta have been plagued by motion artifacts, which can potentially lead to misdiagnoses during the evaluation of aortic dissection. In this study, we have identified a new deep learning-based reconstruction algorithm, which can be applied retrospectively to CT data, has demonstrated remarkable success in effectively reducing motion artifacts of the ascending aorta, thereby providing more acceptable CT images in diagnosing aortic dissection compared to the conventional reconstruction algorithms.

In CT images, the depiction of the aorta is affected by motion artifacts, which can mimic a dissection flap [5]. It has been reported that 30% of patients with an acute aortic dissection diagnosis did not actually have the condition, and all misdiagnoses were secondary to misinterpretation of images with motion artifacts (17%) being common sources of diagnostic error [7]. Researchers have attempted to reduce such artifacts. Takayanagi, et al. reported that using a new-generation CT with an ultrafast scan mode (gantry rotation period of 0.28 s, detector pitch of 1.53, and beam collimation of 80 mm) resulted in a significant reduction of motion artifacts in the ascending aorta compared to the conventional CT (gantry rotation period of 0.4 s, detector pitch of 1.38, and beam collimation of 40 mm) [9]. However, the use of a higher pitch is not without drawbacks. When combined with automatic tube current modulation, it may lead to tube current saturation in weaker X-ray tubes, resulting in deterioration of image quality [11]. Faster CT gantry rotation is also associated with increased image noise [10]. This is why shorter gantry rotation times is not routinely used in our institution. In our study, we retrospectively applied the new-generation image reconstruction algorithm to CT source data acquired with the conventional scan settings (gantry rotation speed of 0.5 s, detector pitch of 0.813, and beam collimation of 40 mm). Our approach does not require electrocardiography triggering. We found that scores of artifacts for the ascending aorta in SR-DLR-M were significantly better than those in SR-DLR by all readers. This indicates the usefulness of motion reduction algorithms during image reconstruction in reducing motion artifacts of the ascending aorta. Improved scores for artifacts have resulted in significantly better image quality, enhancing the acceptability of diagnosing aortic dissection. Furthermore, in patients without aortic dissection, the condition could be more confidently excluded using SR-DLR-M than with SR-DLR.

Another significant impact of this study pertains to the application of SR-DLR. In principle, SR-DLR is anticipated to enhance spatial resolution in comparison to the conventional DLR algorithms. In fact, our results regarding ERS/ERD and subjective sharpness exhibited a significantly improved spatial resolution in SR-DLR-M when compared to DLR-M. Although not theoretically predicted, image noise was also reduced in SR-DLR-M compared to DLR-M, as evidenced by both quantitative and qualitative image evaluations. These results are in line with the previous studies regarding coronary CT angiography, which reported superiority of SR-DLR over DLR in spatial resolution [26] and in image noise [25–27]. The combination of enhanced spatial resolution and diminished image noise would be the primary factors contributing to the significantly enhanced depiction of chest structures and the enhanced acceptability in diagnosing the aortic dissection.

Certain considerations must be taken into account when utilizing SR-DLR-M. First, while the difference may not be substantial, CT attenuation of the structures in SR-DLR-M can exhibit variations compared to that in DLR-M. It has been reported that CT attenuation of the structures can exhibit slight fluctuations based on the reconstruction algorithm (DLR, iterative reconstruction, and filtered back projection) [30–32]. Nagayama, et al. have reported that the coronary attenuation was higher in SR-DLR at distal segments compared to DLR [25], which is also in line with our findings. However, due to the black-box nature of the deep learning techniques, the precise mechanisms underlying this phenomenon remain elusive. Second, image noise in SR-DLR-M was higher than that in SR-DLR, resulting in a significant reduction in the aortic CNR. It is hypothesized that rejecting certain projection data during reconstruction with the motion reduction algorithm (i.e., CLEAR motion technology) may contribute to this phenomenon.

There are some limitations with this study. First, the retrospective nature of this study precludes the recording of heart rate, rendering the correlation between the degree of artifacts and the heart rate unknown. Second, the number of patients with aortic dissection was limited. Given the promising results observed in our study, future investigations that include these patients are warranted. Third, because we applied the new-generation image reconstruction algorithms to CT data acquired with the conventional scan settings, our results may not be directly applied to CT data acquired with reduced radiation dose levels, lower tube voltage, or higher pitch. Fourth, we adopted a reconstruction strength level of 2 for SR-DLR-M, SR-DLR, and DLR-M. The results of our study may not be directly applicable to images reconstructed with different strength levels. Finally, while similar reconstruction algorithms are available from various vendors, the underlying principles may differ across vendors. Consequently, the results may not be directly applicable to CT images acquired from scanners manufactured by other vendors.

In conclusion, SR-DLR-M effectively reduced motion artifacts of the ascending aorta compared to SR-DLR and enhanced the image quality in terms of spatial resolution and image noise relative to DLR-M. These improvements enable the provision of more acceptable chest CT images for the diagnosis of aortic dissection, surpassing those obtained using SR-DLR and DLR-M.
